# Bunyavirus-Vector Interactions

**DOI:** 10.3390/v6114373

**Published:** 2014-11-13

**Authors:** Kate McElroy Horne, Dana L. Vanlandingham

**Affiliations:** 1 Biosecurity Research Institute, Kansas State University, Manhattan, KS 66506, USA; E-Mail: kmhorne@ksu.edu; 2 Department of Diagnostic Medicine and Pathobiology, College of Veterinary Medicine, Kansas State University, Manhattan, KS 66506, USA

**Keywords:** bunyavirus, *Aedes* mosquito, *Culex* mosquito, tick, hemorrhagic fever

## Abstract

The *Bunyaviridae* family is comprised of more than 350 viruses, of which many within the *Hantavirus*, *Orthobunyavirus*, *Nairovirus*, *Tospovirus*, and *Phlebovirus* genera are significant human or agricultural pathogens. The viruses within the *Orthobunyavirus*, *Nairovirus*, and *Phlebovirus* genera are transmitted by hematophagous arthropods, such as mosquitoes, midges, flies, and ticks, and their associated arthropods not only serve as vectors but also as virus reservoirs in many cases. This review presents an overview of several important emerging or re-emerging bunyaviruses and describes what is known about bunyavirus-vector interactions based on epidemiological, ultrastructural, and genetic studies of members of this virus family.

## 1. Introduction to the Bunyaviruses

The family *Bunyaviridae* is the largest RNA virus family, comprised of more than 350 viruses divided into five genera based on serological, morphological, and biochemical characteristics: *Orthobunyavirus*, *Nairovirus*, *Phlebovirus*, *Hantavirus*, and *Tospovirus* with additional unclassified viruses [1]. Viruses within this family, collectively referred to as Bunyaviruses, are all single-stranded, negative sense or ambisense, tripartite RNA genomes with viral genes on one of three segments: the negative sense large (L) segment, coding for the RNA-dependent RNA polymerase for transcription and replication; the negative sense or ambisense (tospovirus) medium (M) segment, which encodes the Gn and Gc viral glycoproteins and a non-structural protein (NSm) of unknown function (possibly virulence; see Rift Valley fever virus discussion in Virus-Vector interactions section), and the small (S) segment, coding for the N nucleocapsid protein and in some genera a non-structural protein (NSs) that may function in the innate immune response, in overlapping or non-overlapping reading frames [2,3,4,5]. The size of viral RNAs and structural proteins, use of ambisense coding strategy, and the identity of consensus sequences at the segment termini differ between genera [5,6,7]. Viruses within the *Orthobunyavirus*, *Nairovirus*, and *Phlebovirus* genera are transmitted by hematophagous arthropods; whereas hantaviruses are transmitted among rodents and tospoviruses are transmitted between plants by non-hematophagous thrips [8,9]. This review will focus on the vector-borne viruses within the *Orthobunyavirus*, *Nairovirus*, and *Phlebovirus* genera. These viruses cause a wide range of clinical syndromes in infected humans and animals, including non-specific febrile illness, encephalitis, and hemorrhagic fever and many species are teratogenic in infected gestating livestock. Selected viruses that have a substantial public health impact as a result of disease in humans and/or animals (listed under “Host(s)”) are presented in Table 1 and discussed in more detail in Section 3.

**Table 1:** Selected medically important vector-borne bunyaviruses.

Genus	Virus	Range	Vector(s)	Host(s)
*Orthobunyavirus*	Akabane virus (AKAV)	Africa, Asia, Australia	Midge	Cattle, sheep, goats
	Cache Valley virus (CVV)	N. America	Mosquito	Sheep
	La Crosse virus (LACV)	N. America	Mosquito	Humans
	Schmallenberg virus (SBV)	Europe	Midges	Cattle, sheep, goats
	Tahyna virus (TAHV)	Europe	Mosquito	Humans
*Nairovirus*	Crimean-Congo Hemorrhagic fever virus (CCHFV)	Europe, Africa, Asia	Tick	Humans
	Nairobi sheep disease virus (NSDV)	Africa, Asia	Mosquito, tick	Sheep, goats
*Phlebovirus*	Rift Valley fever virus (RVFV)	Africa	Mosquito, midge, tick	Humans, cattle, sheep, goats
	Toscana virus (TOSV)	Europe	Sandfly	Humans

## 2. Evolution and Classification

Although viruses in the *Bunyaviridae* family share in common a tripartite RNA genome, they differ greatly in their geographic distribution, disease characteristics, and transmission mode. The family has been divided into the five genera listed above based on antigenic relationships elucidated in a large number of studies reviewed elsewhere [2,10,11]. Notable exceptions to the following exist, but viruses within the *Orthobunyavirus* genus are generally transmitted by mosquitoes, whereas those in the *Nairovirus* genus are primarly tick-borne and viruses in the *Phlebovirus* genus are vectored mainly by sand flies [10]. These vector-virus relationships constrain the geographic distribution and in some cases play a large role in the inter-epidemic maintenance and the evolution of the bunyaviruses. The 2013 release from the International Committee on Taxonomy of Viruses (ICTV) identifies 48 viral species in the *Orthobunyavirus* genus, seven species in the *Nairovirus* genus, and nine species in the *Phlebovirus* genus [12]. Among the orthobunyaviruses, the majority of viruses have been placed into one of 18 serogroups or left unclassified based on antigenic relationships deduced by immunological assays, such as complement fixation or hemagglutination inhibition [2,13]. The 34 recognized nairoviruses have been placed in seven serogroups, corresponding to the seven species recognized by ICTV [14]. The phleboviruses are currently divided into two groups, the sandfly fever group, members of which are all transmitted by mosquitoes and flies, and the tick-borne Uukeniemi-like virus group [6].

Despite the error-prone nature of the viral RNA-dependent RNA polymerase, many arboviruses, including the bunyaviruses remain fairly genetically stable over time due to the requirement that they replicate in both vertebrate and invertebrate cells [10,11,15]. Genetic drift, or the accumulation of minor genetic changes, occurs for bunyaviruses and likely accounts for regional differences observed between some strains of the same virus. In the case of the phlebovirus RVFV, one genetic study identified multiple virus lineages, which correlated with geographic distribution and low genetic diversity of about 5% difference at the nucleotide level among isolates collected between 1951 and 2000 in Africa and the Middle East [16]. However, the major force behind the evolution of bunyaviruses is segment reassortment [17], which is discussed in the context of vector-virus interactions below. Briese *et al.* recently hypothesized that all currently recognized bunyaviruses are reassortants of existing or in some cases extinct viruses [11]. Reassortment events can and have complicated virus identification and classification and can also make characterization of virus epidemiology difficult since serosurveys may not distinguish between viruses with sympatric distribution that share the glycoprotein-encoding M segment [18]. Depending on the identity of the parental viruses and the outcome, reassortment can produce novel viruses with increased pathogenicity or new vector or host range. Several emerging orthobunyaviruses have been identified as reassortants, including recently described Oropouche virus reassortants Jatobal and Iquitos viruses that have been associated with human infections in Brazil and Peru, respectively, and Schmallenberg virus in Europe, discussed further below [11,19,20,21]. Among the phleboviruses and nairoviruses, intratypic reassortment of RVF and Crimean-Congo hemorrhagic fever (CCHF) viruses have been described, and several putative reassortants, such as the phlebovirus Aguacate virus have been identified between known and as yet unidentified viruses [11,16,22,23]. Although natural reassortant viruses clearly have the potential to be substantial public health threats, laboratory generated reassortants have also been useful for assigning function to viral proteins and virus-vector interactions, as described below.

## 3. Selected Medically Important Bunyaviruses

### 3.1. Orthobunyaviruses

The *Orthobunyavirus* genus contains multiple human and animal pathogens in 18 serogroups, including the Bunyamwera, California, and Simbu serogroups, which are transmitted primarily by mosquitoes [18].

#### 3.1.1. Akabane Virus

Akabane virus (AKAV) is a member of the Simbu serogroup that was first isolated from *Aedes vexans* and *Culex tritaeniorhynchus* mosquitoes collected in 1959 in Akabane and other villages in Gunma Prefecture during a long-term study on Japanese arboviral ecology. [24]. The virus is widely distributed throughout Africa, Asia, the Middle East, and Australia, and antigenic and phylogenetic studies have demonstrated substantial strain diversity, even among isolates collected from a single location [25,26,27,28], and differences between phylogenetic trees based on L, M, and S segment sequences suggest that some isolates may be AKAV reassortants [28].

Although adult animals do not usually develop symptomatic infection, AKAV is a significant agricultural pest as it can cause abortions, stillbirth, and congenital deformities, such as arthrogryposis-hydranencephaly syndrome in infected sheep, cattle and goats [25,29]. Before vaccines were developed, AKAV resulted in substantial economic losses in affected regions, including massive losses for the beef and dairy industry in Japan following the birth of over 42,000 abnormal calves from 1972 to 1975 [29]. Commercially available vaccines are attenuated or inactivated variants based on the OBE-1 strain isolated in Japan in 1974, and their widespread use has reduced the incidence of disease [25,28,30,31]. In addition to vaccines, herd management and insect control can be employed but are not effective for long-term control. There have been no reports of human infection with AKAV to date.

Although AKAV has been isolated in mosquitoes, attempts to demonstrate replication in *Cx. tritaeniorhynchus* were unsuccessful [32,33]. The primary vector varies by location and appears to be *Culicoides* midges [28,32], which is consistent with other members of the Simbu serogroup [10]. In Japan where the virus still causes occasional outbreaks, AKAV is vectored by *Culicoides oxystoma*, a midge species that has also been found throughout Asia, Australia, and the Middle East [34,35,36,37]. Pools of other *Culicoides* species, *C. punctuatus*, *C. maculatus*, and *C. jacobsoni*, collected in one Japanese study were negative for AKAV [34]. The virus has also been isolated from *C. imicola* collected in Zimbabwe [38] and Oman [39], but this species has never been collected in Japan [36]. *C. variipennis* was shown to be susceptible to AKAV infection following intrathoracic and oral infection in the laboratory [32,39], and virus was transmitted through a chicken skin membrane when orally infected midges were re-fed 7–10 days post infection in one study. *C. numbeculosus* was susceptible for AKAV following intrathroacic inoculation but not oral infection [32]. The virus is endemic in northern Australia, where the distribution is dependent upon that of its primary vector, *C. brevitarsis*. AKAV has also been isolated in *C. wadai* in Australia, although this species has not been confirmed as a vector [40]. Attempts to detect transovarial transmission (TOT) of AKAV in Australian *C. brevitarsis* were unsuccessful, but the authors postulated that could be due to the low rates of infection of field-collected insects (around 1%) and likely low rate of TOT in an infected vector [40], although there are no published reports describing TOT of AKAV in Australia or anywhere else the virus is transmitted.

#### 3.1.2. Cache Valley Virus

Cache Valley virus (CVV) is a member of the Bunyamwera serogroup that was first isolated in Utah in 1956 from *Culiseta inornata* mosquitoes [18,41]. The virus has been isolated in the United States, Canada, Mexico, Panama, Ecuador, and Jamaica and is regularly detected in serosurveys in North America [18,41,42,43,44,45]. The primary amplifying hosts of CVV are thought to be ungulates, such as deer, sheep, horses, and cattle based on seroprevalence rates, and the virus can cause abortions or congenital malformations in infected sheep [18,41,46,47,48,49]. Although humans in North America have also tested seropositive for CVV or possibly a related virus, there have only been three documented cases of human disease attributed to CVV. The first case in 1995 manifested as severe encephalitis, multi-organ failure, and the eventual death of a previously healthy man following pulmonary complications. The individual reported exposure to mosquito bites in North Carolina two weeks prior to onset of symptoms, and virus was isolated from blood, serum, and cerebrospinal fluid and identified as CVV by electron microscopy and ELISA, followed by confirmation by RT-PCR and sequencing of the S segment [50]. The second case presented as acute aseptic meningitis in a healthy Wisconsin man in 2003 who recovered from the illness, and CVV was confirmed by virus isolation and sequencing of the S segment [46]. The third case involved a 63-year-old woman who initially reported photophobia, neck stiffness, headache and fever and who later recovered. Meningitis caused by CVV was confirmed in this case by qRT-PCR and sequence analysis [45].

The principle insect vector of CVV is unknown [46], but *Anopheles quadrimaculatus* and *Coquillettidia perturbans* have been implicated as vectors in the Midwestern region of the United States [48]. The virus has also been isolated from various other culicine and anopheline mosquitoes, including *Ae. sollicitans*, *Ae. vexans*, *An. punctipennis*, *Cs. inornata*, *Mansonia titillans*, and *Psorophora columbiae* in the U.S., Canada and Mexico in studies conducted between 1965 and 1984 [41]. Reeves and Miller evaluated the vector competence of *C. sonorensis* for CVV, as they are competent transmitters of numerous arboviruses in the U.S. and elsewhere. Only 21% of the midges were infected with CVV, and none of those developed disseminated infections, indicating *C. sonorensis* is not a competent vector, although the authors hypothesized that dual infection with another bunyavirus could result in viral reassortment and the emergence of a virus with altered vector range or pathogenesis [49].

#### 3.1.3. La Crosse Virus

La Crosse virus (LACV) is a member of the California serogroup that first emerged in the United States in the early 1960s [43,51]. The prototype strain was isolated from the brain tissue of a four-year old girl in La Crosse, Wisconsin who died of encephalitis, and the virus is currently the most common mosquito-borne disease of children in the United States, although humans are incidental hosts [51]. Infection may result in encephalitis and aseptic meningitis, and sequelae include learning disabilities and attention deficits [52]. The transmission cycle of this virus has been well characterized and involves *Aedes triseriatus* mosquitoes found in the eastern half of the United States and Southeastern Canada infecting gray squirrels and eastern chipmunks as the primary vertebrate hosts, which do not exhibit signs of clinical infection [4,52]. Sequence differences exist between geographically distant LACV isolates, and LACV reassortment has been documented in nature [53,54]. High rates of LACV transovarial transmission (TOT) have been recorded for *Ae. triseriatus*, which plays a key role in amplification and maintenance of the virus by producing large numbers of LACV infected progeny and eggs in which the virus may overwinter to emerge in the spring [52,55]. Rates of TOT and filial infection can exceed 70%, although infection rates in field-collected materials are often significantly lower [56,57]. LACV is also transmitted horizontally from infected *Ae. triseriatus* males to females, further increasing the number of infected mosquitoes [52,58]. Both *Ae. brelandi* and *Ae. hendersoni*, demonstrate lower transmission rates of LACV in the laboratory than the primary vector due to a salivary gland escape barrier, but the latter is able to transmit the virus transovarially [51,55,59]. *Ae. atropalpus* was also found to transmit LACV orally and transovarially in the laboratory [60]. LACV-infected *Ae. albopictus* have been collected in the field in Tennessee and North Carolina, and *Ae. albopictus* is a highly competent LACV vector in the laboratory that can also transmit LACV transovarially in the lab and field [51,61,62,63]. However, the contribution of this important vector species to LACV epidemiology in the United States is not fully understood.

#### 3.1.4. Schmallenberg Virus

Schmallenberg virus (SBV) was first detected in plasma samples taken from febrile cattle in 2011 in a town in Germany of the same name. It was identified using next-generation sequencing and assigned to the Simbu serogroup. Based on phylogenetic evidence, SBV is most closely related to Sathuperi and Shamonda viruses depending on viral segment [64,65,66] and is hypothesized to be a reassortant of these viruses or a revertant/descendant of Shamonda virus [11,21].

The large 2011 SBV outbreak marked the first known epidemic caused by a member of this serogroup in Europe. Acute infections of adult ruminants or malformed calves, lambs, and goat kids that tested SBV-positive were detected in Germany, the Netherlands, Belgium, France, UK, Italy, Spain, Luxembourg, Denmark and Switzerland during the outbreak from 2010 to 2012 [65]. Fever and decreased milk production have been reported in adult cattle, and SBV pathogenesis of stillborn or malformed ruminant offspring was identical to that described for AKAV [65]. Human infection has not been reported, and no antibodies were detected during serosurveys of humans associated with SBV-positive farms [65,66,67]. Like AKAV, SBV is thought to be transmitted by *Culicoides* midges, as the virus has been detected in members of the *C. obsoletus* group, *C. chiopterus*, *C. dewulfi*, *C. punctatus*, in Belgium, Denmark, Germany, the Netherlands, and Poland [65,68,69,70,71,72]. Colonized *C. sonorensis* developed a disseminated infection following intrathoracic inoculation or oral infection, while *C. nubeculosus* was refractory to SBV infection [64]. Overwintering *An. maculipennis*, *Cs. annulata*, *Cx. pipiens*, and *Cx. territans* mosquitoes tested negative for SBV, and to date the possible role of mosquitoes in SBV epidemiology is unknown [73].

### 3.2. Nairoviruses

The *Nairovirus* genus includes human and animal pathogens that are distinguished from other members of the *Bunyaviridae* family in that they have a very large L segment [14] and are all transmitted by ticks. The genus is composed of seven serogroups based on cross-reactivity profiles, including the Crimean-Congo hemorrhagic fever group, which contains CCHFV and the Nairobi sheep disease group containing Nairobi sheep disease virus (NSDV), both of which are described below [74,75,76].

#### 3.2.1. Crimean-Congo Hemorrhagic Fever Virus

CCHFV is the most widespread tick-borne virus and the most widespread human arboviral pathogen after the dengue viruses [74,76]. CCHFV is also the most genetically diverse of all the arboviruses, displaying 20%–31% sequence homology by segment between isolates [76,77], and intratypic reassortment has been documented for this virus [23]. Although the virus was first recognized as a causative agent of human disease in the former Soviet Union in 1944 and thought to have been circulating for hundreds of years prior, CCHFV was not isolated until the late 1960s after breakthroughs in cultivating and characterizing the virus using suckling mice [76,78]. It has since been discovered that the virus distribution covers much of Europe, Asia, the Middle East and Africa [76]. The name Crimean-Congo reflects the discovery that two separate viruses, Crimean hemorrhagic fever from the Soviet Union and Congo virus from the Congo and Uganda, were antigenically indistinguishable (reviewed by Whitehouse [79]). Many of the original research papers were published in Russian and Bulgarian, but the early research was reviewed extensively by Hoogstraal [78]. Infection in humans ranges from a mild, non-specific febrile illness to severe shock and hemorrhagic syndrome characterized by the development of large ecchymoses, vascular leakage, and multi-organ failure. The case-fatality ratio ranges from 5% in large studies to greater than 30% in some case reports (reviewed by Bente [76]). CCHFV or antibodies have been detected in a variety of domestic and wild animals, including cattle, sheep and goats [76,78,80,81], which may develop viremia lasting up to 14 days but generally show no signs of clinical infection [82]. An inactivated vaccine developed in Russia is used for high-risk populations in Bulgaria, but neutralizing antibody titers generated in response to immunization are low and the only available efficacy information is the observation reduced CCHFV incidence since vaccination was implemented [76,83].

CCHFV is transmitted through the bite of infected ticks or via exposure to blood or bodily fluids of other infected humans or animals. The tick serves as the virus reservoir in nature, as CCHFV is transmitted horizontally and vertically by ixodid (hard) ticks, primarily in the *Hyalomma* genus, which like the virus, have a very wide geographic distribution [76,78]. Although CCHFV has been isolated from two species of argasid (soft) ticks, attempts to develop a persistent infection in soft ticks have been unsuccessful even following intracoelomic inoculation [79,84,85], and isolation from a midge was likely the result of a recent feed rather than a true infection [80]. As reviewed by Bente, Hoogstraal, and Mertens [76,78,86], CCHFV has been isolated from approximately 31 tick species, and implicated vectors include *H. marginatum* in much of Europe and Asia, *H. lusitanicum* in Spain, *H. marginatum rufipes* in Africa, *Rhipicephalus* spp. in the Mediterranean region, and *Dermacentor marginatus* in Turkey. Disease in humans is commonly seen in the spring and summer when the host-seeking activities of adult ticks is highest, and like other arboviruses, known CCHFV distribution mimics that of its tick vectors, particularly that of *Hyalomma* spp.

#### 3.2.2. Nairobi Sheep Disease Virus/Ganjam Virus

NSDV was first identified in sheep in 1910 near Nairobi, Kenya (reviewed by Lasecka and by Yardav [74,87]), and an Asian variant named Ganjam virus was isolated from *Haemaphysalis intermidia* and *H. wellingtoni* ticks and *Cx. vishnui* mosquitoes collected in several locations in India in the late 1960s [88,89,90,91]. The current geographic distribution of the virus includes east and central Africa (NSDV) as well as India (Ganjam). Despite their circulation on different continents, serological cross-reactivity and relatively high sequence identity between NSDV and Ganjam have led to some groups considering the viruses as co-variants rather than distinct species, so both viruses are discussed together in this section [87,92]. NSDV causes severe hemorrhagic gastroenteritis in goats and sheep with a mortality rate approaching 90%, and Ganjam virus is associated with sheep and goats with one report of severe disease in sheep [92]. Serological surveys suggest that human infections with NSDV occur [87], and Ganjam virus has been associated with laboratory-acquired infections [90,93,94]. Human infection is characterized by fever, abdominal and back pain, nausea, and conjunctival infection is seen in some cases [94].

The primary vector of NSDV is *Rhipicephalus appediculatus*, which also transmits the virus transovarially [95,96]. The virus has also been associated with *R. pulchellus* and *A. variegatum* [87,95]. Ganjam virus is primarily associated with *Haemaphysalis intermedia* but has also been isolated from *H. wellingtoni*, *H. parva*, *H. rephiciphalus*, and *R. haemaphysaloides* ticks [87,89,91,97]. Consistent with other arboviruses, virus distribution reflects that of its vector(s).

### 3.3. Phleboviruses

As the name would suggest, a large number of viruses in the *Phlebovirus* genus are associated with *Phlebotomus* sandflies. Other members of the genus, particularly those in the New World, are vectored by *Lutzomyia* spp. sandflies, and some members are mosquito-borne [98]. Interestingly, the most well-known phlebovirus, RVFV, has never been isolated from phlebotomine sandflies in nature, although *P. papatasi*, *P. duboscqi*, *P. sergenti*, *Sergentomyia schweitzi* and *Lutzomyia longipalpus* have been infected with RVFV in the laboratory [99,100,101].

#### 3.3.1. Rift Valley Fever Virus

RVFV was first isolated in the Rift Valley in Kenya in 1931 but was probably responsible for disease in the region since 1910 [3,16]. RVFV endemic circulation is currently limited to Africa and the Middle East, where it causes high rates of abortion and newborn mortality (approaching 100% in some ruminants) and death of domestic and wild animals, including cattle, sheep, and goats [3,16,102]. Humans become infected during animal outbreaks through aerosol or contact with infected animal tissues [3,98,103,104]. Acute infection in humans is characterized by fever, headache and muscle pains, and hemorrhagic manifestations may develop in severe infection with survivors suffering from neurological disorders or vision loss up to several months after infection [105]. In addition to having substantial public health and agricultural impact through infection of humans and animals, RVFV is a Category A priority pathogen in the United States because of the potential ease of dissemination and impact if introduced via natural infection or intentional release [106]. Live-attenuated and inactivated RVFV vaccines exist, including the widely used Smithburn vaccine, which can itself cause abortions if administered to gestating animals. Other vaccines are being developed based on the attenuated MP12 and Clone13 viruses in addition to various subunit candidates, but creating a non-teratogenic vaccine, which elicits rapid and long-lasting immunity without the need for boosters has been a significant challenge [102,107]. The possibilities of teratogenicity and viral reassortment, which has been documented between RVFV strains [16], make use of a live vaccine risky, but inactivated vaccines are expensive to produce and require annual boosters, which can be difficult to implement in the field [102,107].

Like the previously discussed CCHFV, RVFV demonstrates very low vector specificity, reviewed by Pepin *et al.* [102]. The virus is transmitted in nature by greater than 40 mosquito species of the genera *Aedes*, *Culex*, *Anopheles*, *Eretmapodites*, and *Mansonia*, and has also been isolated from *Culicoides* midges, *Simuli* black flies, and *A. variegatum* and *R. appediculatus* ticks [3,102,108,109,110,111] but not sand flies, although this may be due to an underrepresentation of phlebotomines in field-collections [101]. Similar to LACV, mosquitoes play a critical role in the amplification and inter-season maintenance of RVFV through TOT. RVFV can survive for long periods of time in desiccated *Aedes* spp. (including *Ae. circumluteolus*, *Ae. lineatopennis*, *Ae. mcintoshi*, and/or *Ae. vexans*) eggs, which hatch on the edges of damboes during the rainy season to infect nearby humans and animals. Isolation of RVFV from the adult mosquitoes reared from collected larvae and pupae in Kenya supports the possibility of TOT as a mechanism for the maintenance of virus during the inter-epizootic periods [109]. Once the transmission cycle has been initiated, an outbreak may be amplified by *Culex* spp. (including members of the *Cx. pipiens* complex and *Cx. theileri*) mosquitoes [102,109,112]. The association between climate (*i.e.*, rainfall) and RVFV has been incorporated into predictive models for RVFV risk or future outbreaks [107,112] and suggests a role for climate change in propagating virus spread into currently unaffected regions. Several studies have documented vector competence for RVFV in European and North American *Aedes* and *Culex* mosquito species, increasing the possibility of viral spread beyond its current distribution following a natural introduction or an intentional release event [110,113].

#### 3.3.2. Toscana Virus

Toscana virus (TOSV) was first isolated in Italy in 1971 from *P. perniciosus* and *P. perfiliwei* sandflies and has since been detected in many Western European countries, including Portugal, Spain, France, Greece, Croatia, Cyprus and Turkey [114,115]. The virus is a major cause of central nervous system infections during warmer months, when it causes over half of adult hospitalizations for meningitis and meningoencephalitis in central Italy [116]. Seroprevalence rates are high in other areas as well: up to 19% in France [117], 26% in Spain [118], and 20% in Cyprus [119], although some of these results may be attributed to cross-reactivity with related phleboviruses, such as Naples sandfly fever virus [114]. TOSV has been isolated in both female and male sandflies in Italy, Spain, and France, indicating that transovarial and/or venereal transmission, both of which have been demonstrated in laboratory studies of TOSV, occur in nature [114,115]. Serological studies have failed to identify the presence of TOSV antibodies in wild or domestic animals, and the reservoir of TOSV is unknown. Because the virus is transmitted transovarially by phlebotomine flies, these may serve as the viral reservoir, although the rates of vector infection decline through subsequent generations even in the absence of effects on the vector or virus. However, this progressive decline indicates that indefinite TOSV maintenance by the vector is not likely [120,121].

## 4. Vector-Virus Interactions

### 4.1. Insect-Borne Bunyaviruses

It has long been known that in order for a mosquito to transmit an arbovirus, virions in an infectious bloodmeal must infect the midgut epithelium, escape to the hemocoel to infect secondary organs, and be deposited in the saliva when the mosquito takes a subsequent bloodmeal [122]. However, the role of virus, vector, and environmental factors in these processes and the exact mechanisms and route(s) of viral movement through an infected mosquito are less understood. The most well-characterized mosquito-borne bunyavirus with respect to mosquito-virus interactions is LACV in its principal vector, *Ae. triseriatus*, which is known to transmit the virus orally, transovarially, and venereally. Following uptake of an infectious bloodmeal, immunofluorescence studies first detected virus antigen in the pyloric portion of the mosquito midgut, and within seven to 16 days antigen is found in secondary organs, including the heart, neural ganglia, fat body, and the salivary glands and the ovaries, which are most important for viral transmission [4,52,123]. The salivary glands are the last organs to be infected, and virus replicates in this tissue before secretion in the saliva when the mosquito feeds on another host [52]. Virus antigen was present in most organs of both female and male mosquitoes, and venereal transmission was confirmed by detection of LACV in accessory sex gland fluid of infected males [58]. Serial dissection of *Ae. triseriatus* midguts infected with a temperature sensitive mutant of LACV were performed by Sundin and others to track viral antigen abundance and distribution by immunoflourescence assay and titration. While no intracellular antigen was observed four hours post-infection (hpi), antigen was detected in a few cells in the middle of the midgut at 24 hpi then spread throughout the organ and to the cardia and pyloric regions by 48 hpi, and all cells in these regions were involved at 72 hpi, when antigen was also detected in the hindgut. No increase in antigen was detected after this time point, and these results were consistent with viral titers in midguts [124].

In studies on the effect of viral genetics on vector competence, the ability of LACV to infect the mosquito midgut and disseminate to the hemocoel was localized to the Gc glycoprotein on the M segment using a neutralization escape variant, V22, which was markedly attenuated for infection and dissemination in *Ae. triseriatus* following oral infection but equivalent to parental LACV when inoculated intrathoracically, bypassing the midgut [125]. The importance of the M segment of California serogroup viruses in determining vector competence and vector range were confirmed by additional studies in *Ae. triseriatus* and other mosquitoes using California serogroup viruses, described in Section 5. Investigations of the role of environment, in this case nutritional status, on *Ae. triseriatus* competence for LACV found that nutritionally deprived and therefore smaller females had significantly higher rates of viral transmission than their larger, more well-fed counterparts [126]. Further investigation of this effect revealed size-dependent differences in the basal lamina/basement membrane of the midgut in which smaller females had thinner membranes. Given that virions must escape through the basal lamina/basement membrane in order to reach the hemocoel and eventually the salivary glands for transmission, this difference likely accounted for the increase in viral transmission rates and implicated the midgut basal lamina as a physical barrier to viral dissemination within the mosquito [127]. TOT of bunyaviruses, including LACV, is interesting in that in contrast with high viral pathogenicity for developing or young vertebrates, infection of eggs or embryos does not appear to have any deleterious effects on mosquito development. TOT of LACV can affect survival of mosquito eggs, however, as higher embryonic mortality was seen in infected *versus* uninfected eggs following a period of overwintering, although survival rates of infected embryos were still high enough for this mechanism to be effective in maintaining inter-season viral transmission [128]. From the vector genetic side, LACV infection and transmission rates were found to vary greatly between populations collected from different locations, with mosquitoes collected from endemic regions displaying lower viral susceptibility and transmission capability than those collected in non-endemic areas. The authors hypothesized that populations collected in the endemic upper-Midwest were evolving resistance to LACV [129]. Another study identified four mosquito genome regions involved in LACV transmission by mosquitoes using hybrids of the highly competent *Ae. triseriatus* and less competent *Ae. hendersoni*, and three of these regions correlated with body size, possibly confirming the results of the size/competence study by Grimstad *et al.* [127,130]. Studies of RVFV in *Ae. aegypti* and *Cx. quinquefasciatus* identified the functional importance of NSm in virus infection of and escape from the midgut, as mutants deleted for the entire NSm only infected small foci of the *Ae. aegypti* midgut; had reduced infection, dissemination and transmission rates in *Ae. aegypti* compared to wild-type virus; and had lower infection rates in *Cx. quinquefasciatus* than wild-type virus. In contrast, deletion of NSs alone had no effect on *Ae. aegypti* infection or transmission but reduced dissemination of virus [131,132]. Additional studies of RVFV in *Ae. aegypti* (Aag2) and *Ae. albopictus* cell culture (U4.4) determined that the innate antiviral RNA interference (RNAi)-based immune response restricted viral growth and suppressed the formation of NSs filaments in the cells, which allowed RVFV to establish a persistent infection [133]. Whereas experiments using invertebrate cell culture may not be fully representative of virus-vector interactions, these experiments suggested a role for the RNAi response in controlling RVFV infection and allowing the virus to persist in infected cells, which is seen in infected invertebrates in nature. Experiments conducted using Bunyamwera virus in *Ae. albopictus* (C6/36, U4.4, and C7-10) and *Ae. aegypti* (Ae) cell culture found that NSs was essential for replication in U4.4 and Ae cells and in *Ae. aegypti* mosquitoes, suggesting that NSs plays a critical role in virus-mosquito interactions in contrast to data reported by Crabtree *et al.* (2012), but NSs was nonessential for virus replication in C6/36 and C710 cells [134].

Immunofluorescence, immunohistochemistry and electron microscopy have been used to investigate the infection processes and tissue tropisms of RVFV in various studies. Observed barriers to midgut escape and salivary gland infection are thought to result from the presence of the basal lamina, which has pore sizes smaller than that of arboviruses [135]. RVFV infection of midgut epithelial cells was observed to be contiguous in *Cx. pipiens*, in which virus was proposed to spread from cell to cell via the basal lamina [136]. One proposed route of RVFV dissemination from the midgut through the basal lamina was via tracheal cells associated with skeletal muscle and a modified basal lamina, as these were found to be infected in *Cx. pipiens* [135,137]. Virus may also bud directly into the hemolymph from an infected muscle cell [137]. The cardia, composed of midgut tissue at the midgut-foregut junction was also implicated in mediating RVFV amplification and dissemination as it was consistently infected in *Cx. pipiens* with disseminated infections and contains a modified basal lamina that allows RVFV passage [136,137,138]. The fat body, a diffuse tissue found throughout the mosquito, was identified as a site of RVFV amplification [136], and infection of this tissue may mediate a viral titer increase prior to salivary gland infection. Both the lateral and the median lobes of *Cx. pipiens* salivary glands, which are the most important organ in the mosquito for transmission, were infected by RVFV, which is interesting in that only the lateral lobes are infected in other mosquito-virus pairs [136]. Based on studies conducted by his group, Romoser *et al.* proposed the following succession of events during viral dissemination in an infected mosquito: virus infects the midgut epithelial cells to replicate and enter the basal labyrinth. Virus may then infect other epithelial cells, escape through modified basal lamina or an infected tracheal cell, or bud directly into the hemolymph from an infected muscle cell, from where it may disseminate further via the hemolymph or the tracheal system. These routes may explain spurious events of rapid dissemination, in which a tracheal cell is infected very early following uptake of a bloodmeal, and could also account for the consistently observed direct relationship between virus titer in a bloodmeal and mosquito infection rate and subsequent viral transmission in the saliva when the mosquito feeds on a host [137].

The saliva of hematophagous insects and ticks contains factors that assist in bloodmeal delivery and possibly pathogen transmission [139,140], therefore the role of saliva in modulation of viral infection has been investigated for numerous viruses, including the bunyaviruses CVV and RVFV. Edwards and others determined that injection of CVV into sites where *Ae. triseriatus*, *Ae. aegypti* or *Cx. pipiens* have taken a blood meal up to four hours post-feed resulted in viremia and antibody production in mice. No infection was observed when virus was injected alone or at a site distal to the mosquito feed site, indicating that mosquito saliva enhanced CVV infection of mice [141]. Le Coupanec demonstrated increased pathogenicity and mortality in mice when RVFV was delivered with *Ae. aegypti* or *Ae. vexans* saliva or salivary gland extract, but interestingly the same effect was not seen when RVFV was delivered with the salivary gland extracts of *Cx. pipiens* mosquitoes. Comparison of protein composition between species may explain this difference, and the authors hypothesized that identification and characterization of any potentiating factors in mosquito saliva could facilitate the development of novel prophylactics or therapeutics for mosquito-borne viral infections [142].

### 4.2. Tick-Borne Bunyaviruses

Like hematophagous insects, female ticks must take a bloodmeal in order to obtain nutrients for oogenesis. Ticks also take blood from a vertebrate in order to obtain nutrients for metamorphosis to subsequent instars (life stages, larva to nymph to adult), and ticks remain infected for life. Many species of ticks take multiple bloodmeals on different hosts, choosing a new host for different instars, which can affect virus circulation [143]. Relatively little is known about the interactions between tick-borne viruses and their vectors however. In addition to the fact that ticks can be difficult to work with in the laboratory, the most epidemiologically significant tick-borne virus, CCHFV, must be studied under BSL-4 containment, which limits the available facilities and personnel for research on the virus. Even less is understood about the interaction between RVFV and ticks beyond the fact that RVFV has been isolated in *A. variegatum and R. appediculatus*.

Although CCHFV has been isolated from numerous hard and soft tick species, *Hyalomma* spp. ticks are thought to be the most important for CCHFV epidemiology [79,85]. Isolation of virus in a tick is not sufficient to implicate a species as a vector since virus may be contained in a recent bloodmeal rather than tick tissues. Shepherd *et al.* concluded after studies of CCHFV replication in and transmission by various ixodid (hard) and argasid (soft) tick species that ixodid ticks are the true vectors of CCHFV. *H. m. rufipes*, *H. truncatum*, *Rhipicephalus evertsi mimeticus* maintained virus infection up to 205 days post-intracoelomic inoculation, and adult ticks transmitted CCHFV to sheep, whereas argasid ticks were not susceptible to infection [81]. Virus titers in male and female ticks were the same following intracoelomic inoculation of CCHFV, and infection rates and titers in salivary glands, ovaries and testes increased upon blood feeding [144]. Following oral ingestion of a bloodmeal, virus infects and replicates in the midgut of the tick, then disseminates to the hemolymph and subsequently infects the salivary glands [144]. Like mosquitoes, ticks differ in the minimum threshold of virus titer necessary to infect the midgut [145]. Transovarial, transstadial (between life stages), and venereal transmission has been documented in the laboratory or field among *Hyalomma* and *Dermacentor* spp. ticks (reviewed by Whitehouse and by Bente *et al.* [76,79]), implicating the tick as a reservoir of CCHFV as well as the vector. Ticks can also transmit virus “non-viremically,” directly to ticks feeding in close proximity on the same animal. It is thought that this mechanism of transmission is enhanced by components of tick saliva [85], and this can also enhance TOT [143,146]. Because each female tick can produce thousands of eggs, TOT is critical to virus maintenance, but unfortunately not much is known about the viral, vector, and environmental factors that affect tick-virus interactions.

## 5. Viral Reassortment

Bunyaviruses are relatively unique among arboviruses in their ability to rapidly and dramatically evolve by reassortment of their segmented genomes following concurrent infection of a single cell with two viruses. This may occur between homotypic (same species) or in some cases heterotypic (same genera) viruses with sympatric distribution and a shared susceptible vector [2,4,11,52]. As noted above, reassortment between the same or similar viruses has been documented in nature for the *Orthobunyavirus*, *Nairovirus*, and *Phlebovirus* genera and may result in the emergence of a previously unrecognized viral species as has been hypothesized for SBV, and may result in the emergence of a virulent virus, such as Ngari virus, a reassortant of Bunyamwera and Batai viruses [147]. The vast majority of putative reassortant viruses are mosquito- and culicoid-borne rather than tick-borne, and this is likely due to differences in feeding patterns between dipterans, which feed frequently, and ticks, which are long-lived but feed infrequently, decreasing the chances of dual infection [11]. Although other segmented viruses, such as the influenza virus are able to undergo reassortment in vertebrate hosts, it is unknown whether this occurs with bunyaviruses so will not be discussed in this review [4,148]. Therefore, the focus of this section will be upon reassortment in the mosquito, in which the majority of reassortment studies have been conducted.

For reassortment in the mosquito vector to occur, mosquitoes must become infected with two different viruses, and there are several mechanisms by which this may occur in nature. First of all, a mosquito may imbibe the blood of two hosts infected with different viruses, either by taking more than one bloodmeal in her lifetime or by interrupted feeding (partial engorgement from one host that is interrupted followed by completion of feeding on another). TOT of several bunyaviruses has been documented and could result in dual infection if a female that has been transovarially infected (TI+) with one virus takes a viremic bloodmeal during the course of her lifetime [4,149]. One factor that may limit the incidence of reassortment, is the phenomenon of superinfection resistance, which has been well-studied in bunyaviruses. Understanding the timing and mechanisms is important since hematophagous arthropods may take several bloodmeals within their lifespan, increasing the chances of dual viral infection and therefore reassortment. Although interference to superinfection of mosquitoes with another bunyavirus occurs within several days and increases over time [148,149], mosquitoes that are transovarially infected with one bunyavirus may become infected with another. This is probably due to the fact that TI+ mosquitoes typically have much lower virus titers in the midgut than mosquitoes that have been orally infected with the same virus [2,52,149,150]. Venereal transmission of virus is another mechanism that has been observed for some bunyaviruses, such as LACV or Tahyna virus (TAHV), another orthobunyavirus in the California serogroup, which could result in dual infection. A male mosquito that has been transovarially infected with one virus may inseminate a female infected by a heterologous virus, resulting in dual infection of the female mosquito [52,58].

Numerous studies have been conducted to evaluate California serogroup [LACV, TAHV, Jamestown Canyon virus (JCV), and Snowshoe hare virus (SSHV)] reassortants in mosquitoes, primarily *Ae. triseriatus* due to past difficulty in colonizing other mosquito species. In the absence of now widely available genotyping techniques, many studies used temperature sensitive (ts) mutants [151,152]. Beaty and others used wild type and ts LACV to demonstrate high rates of dual infection following simultaneous infection or interrupted feeding. They found that the incidence of dual infection decreased over time due to superinfection resistance, and reassortants were generated in *Ae. triseriatus* [148,153]. The majority of studies demonstrate the importance of the glycoprotein-encoding M segment in transmission and vector range. A strong correlation was found between the LACV M segment and transmission of virus to suckling mice in experiments utilizing LACV and SSH reassortants and *Ae. triseriatus*. Transmission rates were 90% for reassortants with LACV M *versus* 35% for reassortants with SSHV M segment. This was postulated to be a barrier at the salivary glands [153,154], which is not surprising given that *Ae. communis* and *Cs. inornata* are the natural vectors of SSHV [4]. A later study determined that LAC-SSHV reassortant viruses were transmitted vertically and horizontally following intrathoracic inoculation of parental viruses [151]. Cheng *et al*, found that reassortants between LACV and JCV were able to infect *Ae. triseriatus* orally only when they contained the LACV M segment, which is significant in that JCV is not vectored in nature by *Ae. triseriatus*. All reassortants infected and were transmitted by *Ae. albopictus*, which again mirrors the natural vector range of LACV and JCV [155]. For LACV and TAHV, a high frequency of reassortment (42%) was found to occur in *Ae. triseriatus* inoculated with the two viruses. The same study showed by chi-squared analysis that the segment combinations following co-infection of *Ae. triseriatus* mosquitoes with LACV and TAHV were not generated completely randomly. All possible combinations of reassortants were generated in this study; 73% of the new viruses contained the LACV L segment while 79% contained the TAHV S segment. The M segment distribution was random at 45% LACV and 55% TAHV [150]. The significance of this distribution was not discussed, but it is interesting to note that the segment most commonly associated with vector range and virulence, the M segment, was distributed approximately equally. Non-random segment distribution after reassortment was also documented between Batai, Bunyamwera, and Maguari viruses, for which cosegregation of the parental L and S segments occurred in reassortant progeny [156].

## 6. Conclusions

Research on bunyaviruses has contributed a wealth of basic information about vector-virus interactions to the field of arbovirology, although there are still many areas for which sufficient data has yet to be collected. It is clear that bunyaviruses continue to pose a significant threat to human and agricultural public health. Promising areas for additional research on the viruses covered in this review and other medically important bunyaviruses, such as Oropouche virus, include further characterization of the genetic determinants of virus infection, dissemination and transmission by insect and tick vectors and mechanisms for blocking viral infection or transmission by these vectors. Because many bunyaviruses use the vector as a mode of transmission as well as a reservoir, elimination of many of these viruses will be impossible. Therefore, the development of new or additional human and agricultural vaccines to mitigate disease incidence in endemic areas, which include much of the globe when the *Bunyaviridae* family as a whole is considered, should be considered a priority for those pathogens detailed in this review and additional emerging viruses as necessary.
